# Lipopolysaccharide-induced neuroinflammation induces presynaptic disruption through a direct action on brain tissue involving microglia-derived interleukin 1 beta

**DOI:** 10.1186/s12974-019-1490-8

**Published:** 2019-05-18

**Authors:** Olivia Sheppard, Michael P. Coleman, Claire S. Durrant

**Affiliations:** 1 John van Geest Centre for Brain Repair, Department of Clinical Neurosciences, University of Cambridge, E.D Adrian Building, Forvie Site, Robinson Way, Cambridge, CB2 0PY UK; 2 Signalling Programme, Babraham Institute, Babraham Research Campus, Cambridge, CB22 3AT UK

**Keywords:** Organotypic hippocampal slice culture, Lipopolysaccharide, Microglia, IL1β, Synapse, Synaptophysin, Presynaptic, Alzheimer’s disease

## Abstract

**Background:**

Systemic inflammation has been linked to synapse loss and cognitive decline in human patients and animal models. A role for microglial release of pro-inflammatory cytokines has been proposed based on in vivo and primary culture studies. However, mechanisms are hard to study in vivo as specific microglial ablation is challenging and the extracellular fluid cannot be sampled without invasive methods. Primary cultures have different limitations as the intricate multicellular architecture in the brain is not fully reproduced. It is essential to confirm proposed brain-specific mechanisms of inflammatory synapse loss directly in brain tissue. Organotypic hippocampal slice cultures (OHSCs) retain much of the in vivo neuronal architecture, synaptic connections and diversity of cell types whilst providing convenient access to manipulate and sample the culture medium and observe cellular reactions.

**Methods:**

OHSCs were generated from P6-P9 C57BL/6 mice. Inflammation was induced via addition of lipopolysaccharide (LPS), and cultures were analysed for changes in synaptic proteins, gene expression and protein secretion. Microglia were selectively depleted using clodronate, and the effect of IL1β was assessed using a specific neutralising monoclonal antibody.

**Results:**

LPS treatment induced loss of the presynaptic protein synaptophysin without altering PSD95 or Aβ protein levels. Depletion of microglia *prior to* LPS application prevented the loss of synaptophysin, whilst microglia depletion *after* the inflammatory insult was partially effective, although less so than pre-emptive treatment, indicating a time-critical window in which microglia can induce synaptic damage. IL1β protein and mRNA were increased after LPS addition, with these effects also prevented by microglia depletion. Direct application of IL1β to OHSCs resulted in synaptophysin loss whilst pre-treatment with IL1β neutralising antibody prior to LPS addition prevented a significant loss of synaptophysin but may also impact basal synaptic levels.

**Conclusions:**

The loss of synaptophysin in this system confirms LPS can act directly within brain tissue to disrupt synapses, and we show that microglia are the relevant cellular target when all major CNS cell types are present. By overcoming limitations of primary culture and in vivo work, our study strengthens the evidence for a key role of microglia-derived IL1β in synaptic dysfunction after inflammatory insult.

**Electronic supplementary material:**

The online version of this article (10.1186/s12974-019-1490-8) contains supplementary material, which is available to authorized users.

## Background

Neuroinflammation has been linked to synapse loss and cognitive decline both in humans and in pre-clinical models, but important questions remain about the cellular mechanisms that existing experimental systems cannot easily address. Systemic inflammatory events such as sepsis, periodontitis, infections, bone fracture and post-operative trauma can result in sustained high levels of circulating pro-inflammatory cytokines [1, 2] and have been linked with long-term cognitive impairment in patients [3–7]. Observations that systemic inflammation accelerates cognitive decline in Alzheimer’s disease [2, 8–10] as well as exacerbating disease processes in other neurodegenerative disorders [11–13] mean that understanding how inflammatory processes result in synapse loss in the brain is a key requirement for designing effective therapeutics.

Studies investigating the link between systemic inflammation and changes in the central nervous system (CNS) in vivo induce systemic inflammation either aseptically (via administration of lipopolysaccharide (LPS), a potent endotoxin found on the cell walls of gram-negative bacteria [14]) or by inducing sepsis for example via cecal ligation and puncture [15, 16]. Such studies have suggested that induction of systemic inflammation can result in the activation of microglia, increased production of pro-inflammatory cytokines such as interleukin 1-beta (IL-1β) and loss of synaptic proteins in the hippocampus which coincides with cognitive impairment [16–23]. Links between mechanisms of synapse loss in Alzheimer’s disease and systemic inflammation have also been explored, with the contradictory findings that LPS administration can increase the production of Aβ [20, 24, 25] but results in enhanced clearance of diffuse plaques from mouse models of Alzheimer’s disease [26–29]. Whilst in vivo models have proved useful to model aspects of neuroinflammatory processes, mechanistic studies exploring how inflammatory insults lead to synaptic alterations in the brain are constrained by difficulty in determining which steps take place within the periphery and which within the brain, by breakdown of the blood-brain barrier and by limited control over experimental conditions and observations. Proving the pivotal role of microglia in vivo, for example, is challenging, as depletion of microglia, or inhibition of activation, may also target invading peripheral immune cells [30]. Similarly, whilst there are reports of increased IL1β production in the hippocampus after microglial activation [15, 16, 18, 19, 23], determining whether such a change is directly responsible for synaptic deficits is complicated by difficulty in targeting IL1β antagonising drugs or neutralising antibodies to the brain and maintaining their levels [31, 32]. As such, many studies seek to explore proposed mechanisms using primary culture models.

Applying conditioned medium from LPS-activated microglia cultures to primary hippocampal neurons has been shown to induce loss of synapses, with this effect attenuated by the co-application of IL1-receptor antagonists or IL1β-neutralising antibodies [16, 33]. Similarly, direct application of IL1β to primary neuronal cultures results in loss of synapses [16, 33, 34] and depresses synaptic transmission [35]. However, whilst these studies provide insight into how microglial activation may result in synaptic disruption, such systems are, by necessity, an over-simplification of the CNS environment. Synapses formed in primary neuronal cultures will differ from those found in vivo, which form as part of highly structured connectivity networks and are heavily influenced by resident glial cells [36–39]. Astrocytes, for example, have been shown to be involved in the development, support and elimination of synapses [40, 41], and cross-talk between neurons in functional circuits regulates synaptic strength and plasticity [42]. Physically isolating microglia from neurons in such studies also prevents consideration of the role direct contact between these cell types play in the formation, maintenance and destruction of synapses under physiological and pathological conditions [43–46]. Studying microglial responses in isolation from other glial cells could also mask relevant phenotypes. For example, whilst conditioned medium from LPS-stimulated microglia resulted in the loss of synapses when applied to neuronal culture, treatment with LPS-stimulated astrocyte-conditioned medium increased synapse formation [16]. Co-culture of astrocytes and microglia has also been found to alter the response to LPS [47]. Taken together, it is apparent that there are gaps in the mechanistic exploration of inflammation-induced synaptic disruption that neither primary culture, in vivo studies, nor even combinations of the two, can address.

Organotypic hippocampal slice cultures (OHSCs), where thin sections of the hippocampus are maintained in culture for several weeks [48–50], represent a crucial intermediate between in vivo and primary culture models and offer an excellent opportunity to explore mechanisms of synaptic disruption in neuroinflammation. Slice cultures retain hippocampal cytoarchitecture, synaptic connections and populations of supporting cell types in a system isolated from peripheral confounds that is amenable to experimental manipulation and observation [48–51]. As multiple slices can be produced from the same animal, experimental treatments can also be compared to controls from the same biological sample, reducing the number of animals required for effective experimentation, and ensuring baseline differences between animals do not mask the effects of experimental manipulations [50].

In this study, we retest the hypotheses that LPS treatment can disrupt synapses through a direct action on brain tissue, that this is dependent on microglia and IL1β, and we test whether such synaptic disruption is reversible. We confirm that this occurs in the absence of significant neuronal cell loss or alterations in Aβ production. This work highlights a key role of microglial-derived IL1β in neuroinflammatory synapse loss and tests the potential for therapeutic intervention and recovery.

## Methods

### Mice

Wild-type mouse pups (C57BL/6Babr), at age 6–9 days old were obtained from the breeding colony at the Babraham Institute. Animal work was approved by the Babraham Institute Animal Welfare, Experimentation and Ethics Committee and was performed in accordance with the Animals (Scientific Procedures) Act 1986 under Project License PPL 70/7620 and P98A03BF9. All animals were bred in a specific pathogen-free animal facility with strict temperature and humidity control. Both genders were used in experiments.

### Organotypic hippocampal slice cultures

OHSCs were cultured according to the interface method as described previously [48, 49]. Briefly, P6-P9 mouse pups were humanely sacrificed by cervical dislocation and their brains rapidly transferred to ice-cold dissection medium (EBSS + 25 mM HEPES +1× penicillin/streptomycin). Brains were transected at the midline and glued (Loctite) to a vibratome stage. Three hundred fifty-micrometre sagittal slices were cut using a Leica VT1000S vibratome, and the hippocampus and associated entorhinal cortex dissected out. Slices were plated on 0.4-μm pore membranes (Millipore: PICM0RG50) sitting on top of 1 ml of maintenance medium (50% MEM with Glutamax-1 (Life Tech: 42360-024), 25% heat-inactivated horse serum (Life Tech: 26050-070), 23% EBSS (Life Tech: 24010-043), 0.65% d-glucose (Sigma: G8270), 2% penicillin-streptomycin (Life Tech: 15140-122) and 6 units/ml Nystatin (Sigma: N1638)). Two to four culture dishes per pup were made, depending on the experimental protocol, with two to three slices plated per dish. One and 4 days after plating, cultures underwent a 100% medium exchange, before moving to a 50% weekly exchange thereafter. Cultures were maintained in an incubator under high humidity at 37 °C and 5% CO_2_ for up to 5 weeks.

### Treatments

At 2 weeks in vitro, OHSCs were treated with 200 ng/ml lipopolysaccharide from *Escherichia coli* O55:B5 (LPS) (Sigma: L5418) or 20 ng/ml murine interleukin-1β (IL-1β) (Sigma: I9401) for an additional 7 days. For microglial depletion experiments, OHSCs were treated with 100 μg/ml clodronate (VWR: 233183) for 24 h prior or 24 h after, and throughout, LPS treatment. For IL1β-neutralising experiments, OHSCs were pre-treated with either 1 μg/ml murine-IL1β-neutralising mouse monoclonal antibody (Invivogen: mabg-mil1b) or 1 μg/ml mouse IgG isotype control antibody specific to *E. coli* β-galactosidase (Invivogen: mabg1-ctrlm) for 24 h prior and throughout LPS treatment. To assess cell death, OHSCs were incubated with 1 μg/ml propidium iodide (ThermoFisher Scientific: P3566) for 15 min, washed in EBSS then imaged using a DMi8 Leica fluorescence microscope.

### Western blotting

OHSCs were scraped off the membrane into ice-cold RIPA buffer (50 mM Tris-HCl, 500 mM NaCl, 1% Triton-X, 10 nM EDTA, pH 8.0) with protease and phosphatase inhibitors (ThermoFisher Scientific: 78442). Slices underwent probe sonication for 2 × 5 s to completely homogenise the tissue. Equal amounts of protein were denatured in Laemelli buffer (with 2-Mercaptoethanol) and loaded into 4–20% Tris-glycine gels for separation by SDS-PAGE. Proteins were transferred onto PDVF-FL prior to blocking in Odyssey blocking buffer for 1 h at room temperature. Primary antibodies were diluted in 5% BSA in PBS-T with 0.05% sodium azide, and membranes were incubated overnight at 4 °C on the shaker. After 3 PBS-T washes, membranes were incubated in 1:10,000 secondary IRDye anti-mouse and anti-rabbit antibodies (Li-Cor) for 2 h (protected from light), washed with PBS-T then imaged using a Li-Cor Odyssey CLX system. Band intensities were normalised to beta iii tubulin (Tuj1) to control for differences in neuron number. Primary antibodies were used as follows: 1:1000 mouse synaptophysin (Abcam: ab8049), 1:500 rabbit PSD95 (Abcam: ab18258) and 1:2500 rabbit Tuj1 (Sigma: T2200).

### Immunohistochemistry

Slices remained adhered to membranes whilst fixed for 20 min in 4% PFA and then washed three times in PBS. The membranes were then cut, and slices were placed in a 24-well plate and blocked for 1 h in blocking solution (PBS + 0.5% Triton X-100 + 3% goat serum). Slices were incubated with primary antibody (1:500 Iba-1 (Alpha Laboratories: 019-19741)) diluted in blocking solution overnight with shaking at 4 °C. After three PBS washes, OHSCs were incubated (2 h, room temperature, protected from light) with Alexa488 or 568 conjugated secondary antibodies (Life Technologies) diluted 1:250 in blocking solution. Slices were counterstained with Hoechst (1:5000 in PBS), washed in PBS then mounted on slides to be imaged via confocal microscopy. Iba1 coverage was assessed via ImageJ, with the area of Iba1 immunostaining expressed as a percentage of the total image area.

### Quantitative PCR

RNA was extracted from OHSCs using the RNEasy Extraction Kit (Qiagen: 74104). From this, cDNA was synthesised using a Reverse Transcriptase Kit (Quantitect: 205310). Quantitative PCR was carried out using Brilliant III Ultra-Fast SYBR Green QPCR Master Mix (Agilent Technologies: 600882). The following PCR programme was used on a BIO-RAD CFX96 Real-Time PCR Detection System with c1000 Touch Thermal cycler: 3 min at 95 °C, 40 cycles of 5 seconds at 95 °C and 5 seconds at 60 °C. Primers for each gene are listed in the table below.

**Table:** 

Gene name	Forward primer (5′-3′)	Reverse primer (5′-3′)
Pgk1	CTATCATAGGTGGTGGAGAC	ACACTAGGTTGACTTAGGAG
Hprt	AGGGATTTGAATCACGTTTG	TTTACTGGCAACATCAACAG
Ywhaz	ACTTAACATTGTGGACATCG	GGATGACAAATGGTCTACTG
Synaptophysin (SYP)	GATGTAATCTGGTCAGTGAAG	TAGGGCTCAGACAGATAAATAG
PSD95 (dlg4)	ATTGGAAAGGGGTAACTCAG	CTTGGTGATAAAGATGGATGG
IL1β	GGATGATGATGATAACTGC	CATGGAGAATATCACTTGTTGG
APP	CAAAAACTGGTGTTCTTTGC	TGATGGATGGATGTGTACTG

### ELISA

To determine the level of murine Aβ_1–42_ or IL1β in the culture medium, ELISAs were carried out using commercially available kits (Invitrogen: KMB3441 or R&D Systems: MLB00C). Medium was collected from slice cultures at various timepoints throughout LPS or IL-1β treatment. ELISA was carried out as per the manufacturer’s instructions, with absorbance read using a PheraStar FS plate reader.

### Statistical analysis

Data was analysed using GraphPad Prism Software. Statistical tests were chosen to match the data set type, including paired and unpaired *T* tests and two-way ANOVA. Significance values are reported as follows: *p* < 0.05*, *p* < 0.01**, *p* < 0.001*** and *p* < 0.0001***. Error bars are mean ± SEM.

## Results

### LPS treatment induces the loss of the presynaptic protein synaptophysin

**Fig. 1:**
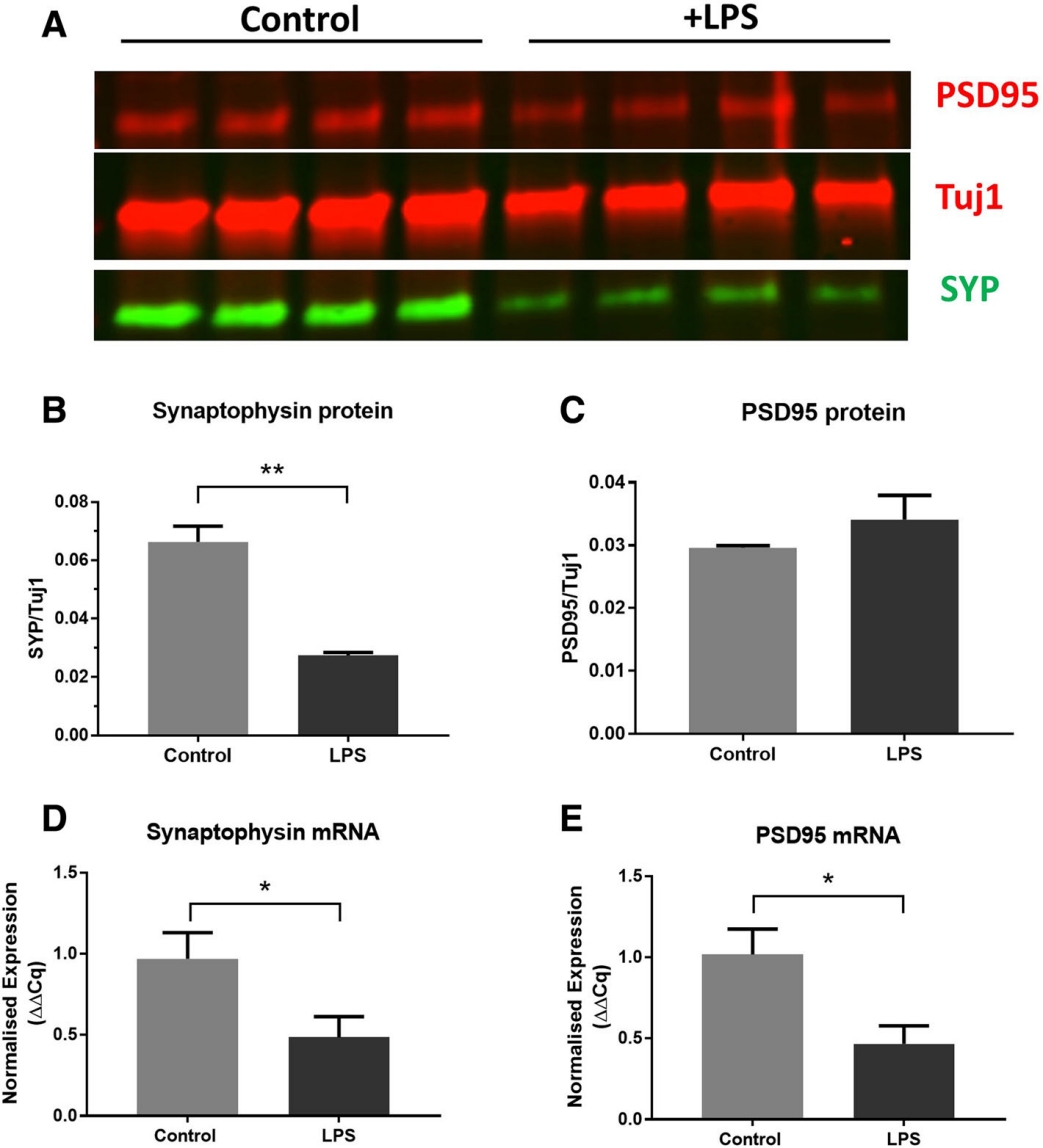
LPS addition causes reduction of synaptophysin protein with no change in PSD95. Fourteen days in vitro organotypic hippocampal slice cultures were challenged with 200 ng/ml LPS for a further 7 days. Slice cultures were harvested, and synaptic proteins examined by western blot (**a**). Seven days of LPS treatment results in loss of the presynaptic protein synaptophysin (**p = 0.0051) (**b**) but with no change in the post-synaptic protein PSD95 (*p* = 0.32) (**c**) (*n* = 4 per treatment group). There is a significant decrease in synaptophysin mRNA (*p = 0.014) (**d**) and PSD95 mRNA (*p = 0.012) (**e**) after LPS treatment (*n* = 10 per treatment group). All statistics were conducted using a paired *t* test to account for matched control and treated OHSCs from the same animal. Error bars = mean ± SEM

OHSCs were created from P6-P9 wild-type mice such that two separate culture dishes (each with three hippocampal slices) were generated per animal. Cultures were aged for 14 days in vitro before treatment with 200 ng/ml LPS. Slices were collected for western blot or qPCR analysis after 7 days of treatment. For all analysis, LPS-treated cultures were directly compared to the untreated control from the same animal. Figure 1a shows a representative western blot where lysates were probed for the presynaptic protein synaptophysin (SYP), post-synaptic protein PSD95 and neuronal microtubule protein beta iii tubulin (Tuj1). Synaptic protein levels were normalised to Tuj1, in order to control for any loss of neurons that may confound any specific vulnerability of the synapses, and propidium iodide staining confirmed that cell death after LPS treatment was minimal (Additional file 1: Figure S 1). LPS treatment resulted in a significant loss of synaptophysin (**p = 0.0051) (Fig. 1b) but did not alter the levels of PSD95 (*p* = 0.32) **(**Fig. 1c). Interestingly, qPCR analysis revealed a significant decline in both synaptophysin (Fig. 1d) (*p = 0.014) and PSD95 (Fig. 1e) (*p = 0.012) transcript relative to three housekeeping genes (Pgk1, Ywhaz, Hprt). The decline in PSD95 transcript may predict eventual protein decline on the post-synaptic side. Thus, LPS applied directly to isolated brain tissue disrupts synaptic protein content with presynaptic changes preceding those on the post-synaptic side.

### LPS-induced synaptophysin loss is microglial dependent

**Fig. 2:**
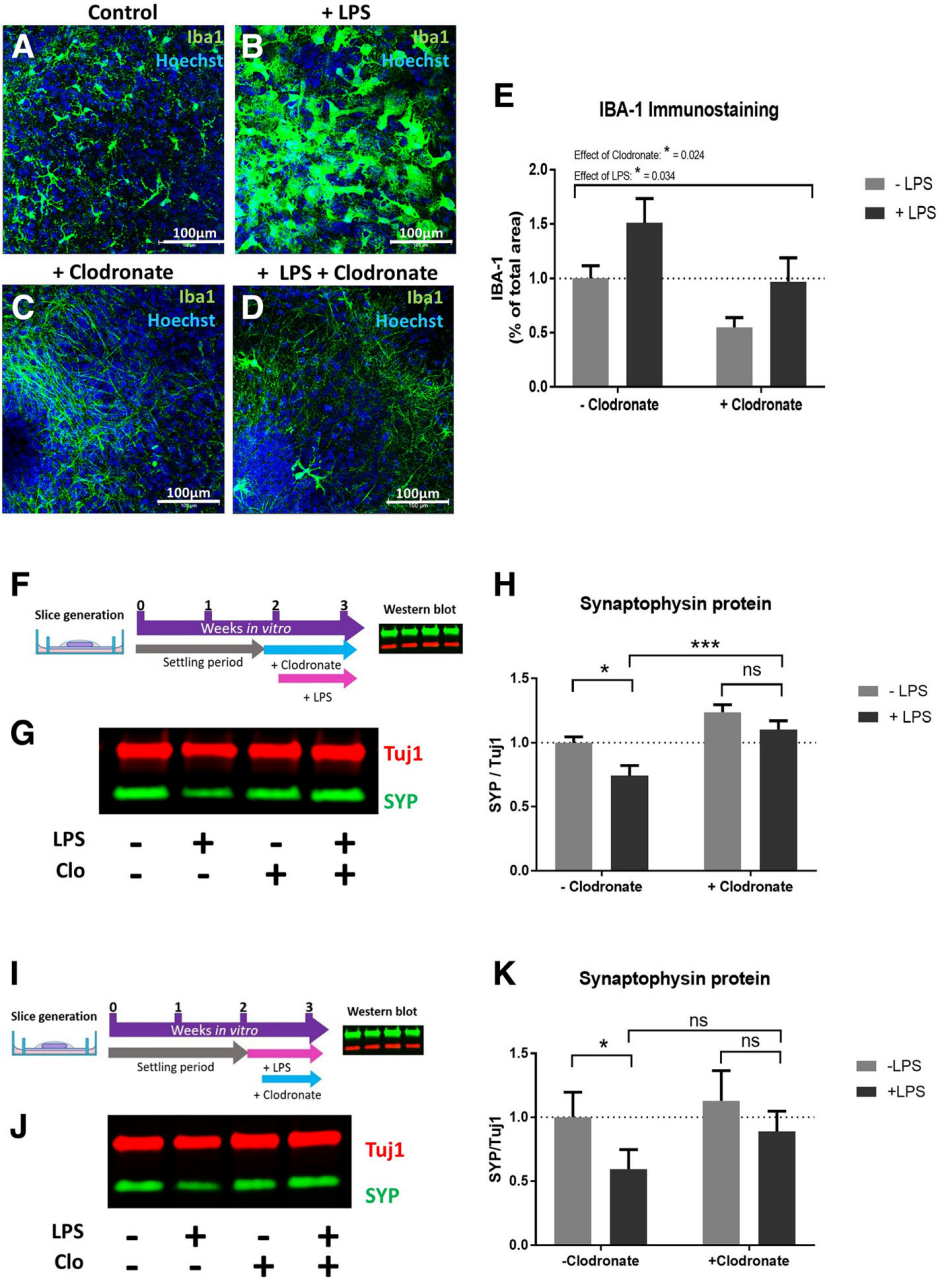
Pre-treatment with clodronate selectively kills microglia and prevents LPS-induced synaptophysin loss. **a**–**d** Immunostaining of LPS and clodronate-treated OHSCs (green = Iba1, blue = Hoechst). Whilst microglia in control conditions appear ramified (**a**), addition of LPS results in a striking alteration of microglial phenotype to an amoeboid morphology (**b**). Pre-treatment of OHSCs with clodronate significantly reduces the number of recognisable microglia in LPS-naïve cultures (**c**) and LPS-exposed cultures (**d**). There is an overall reduction in the area of Iba1 immunostaining after clodronate treatment (*p = 0.024) with an overall effect of LPS to increase Iba1 coverage in OHSCs (*p = 0.034) (**e**) (*n* = 4–11 per treatment group). Schematic showing experimental schedule for clodronate pre-treatment (**f**). Western blot of LPS and clodronate-treated cultures (**g**, **h**) shows that whilst clodronate-naïve cultures show a reduction in synaptophysin when treated with LPS (*p = 0.03) there is no difference between clodronate pre-treated cultures upon additional LPS application (*p* = 0.47). There is a significant rescue seen when comparing the effect of clodronate pre-treatment on cultures treated with LPS (***p = 0.0009). There is a significant overall effect of clodronate treatment regardless of LPS addition (*p* ≤ 0.0001****) (*n* = 20 per treatment group). Schematic of experimental protocol for clodronate application after LPS treatment (**i**). Western blot (**j**) shows clodronate-naïve cultures show a loss of synaptophysin when exposed to LPS (*p = 0.019) whereas there is no difference between clodronate alone versus LPS + clodronate cultures (*p* = 0.24) (**k**). There is, however, no significant rescue when comparing the presence or absence of clodronate in LPS-treated cultures (*p* = 0.11). There is a significant effect of clodronate regardless of LPS treatment (*p = 0.036) (*n* = 18 per treatment group). All statistics were conducted using a two-way ANOVA. Error bars = mean ± SEM

To test whether microglia mediate the effect of LPS directly on brain tissue, these cells were depleted from slice cultures using the specific toxin clodronate [30, 52, 53] at 13 days in vitro. Twenty-four hours later, half of the cultures received 200 ng/ml LPS resulting in four treatment groups: control (no treatments), LPS only, clodronate only and clodronate + LPS. Cultures were harvested at 21 days in vitro (7 days after LPS treatment) (Fig. 2f). Immunofluorescence staining for the microglial marker Iba1 revealed a change in morphology and increase in Iba1 area after LPS addition (Fig. 2a–e). Whilst the microglia detected in untreated (control) OHSCs were in a ramified, branched state (Fig. 2a), LPS treatment (in the absence of clodronate) resulted in an increase in total area of Iba1 signal as well as a shift to an amoeboid morphology (Fig. 2b). Pre-treatment with the microglial toxin clodronate resulted in almost complete depletion of recognisable microglia, even in the presence of LPS (Fig. 2c, d), even though some narrow processes remained. The effect on LPS-induced synaptophysin loss was assessed by western blot (Fig. 2g) with the results showing that pre-treatment with clodronate before LPS addition could block the loss of synaptophysin protein (Fig. 2h). Whilst OHSCs treated with LPS in the absence of clodronate showed a significant reduction in synaptophysin (*p = 0.03), there was no difference between clodronate-treated and clodronate + LPS-treated synaptophysin levels (*p* = 0.47). There was a significant rescue of synaptophysin levels when comparing LPS-treated to clodronate + LPS-treated cultures (***p < 0.001). This rescue indicates a role of OHSC microglia in the effect of LPS on presynaptic proteins in a system where any effects of clodronate on peripheral cell types [30] can be excluded. It is interesting to note that as well as preventing the LPS-induced synaptophysin loss, the addition of clodronate regardless of LPS treatment increased the levels of synaptophysin protein (two-way ANOVA effect of clodronate ****p < 0.0001) suggesting a role for microglia in regulating basal levels of synaptic protein in our OHSC model.

To examine whether synaptophysin loss could be prevented by targeting microglia *after* application of the inflammatory insult, to model a clinical situation where inflammation is treated after it has already occurred, clodronate was applied 24 h after LPS addition, and cultures harvested after a further 6 days in vitro (7 days after LPS application) (Fig. 2i). As before, western blot analysis (Fig. 2j) showed that there was a significant depletion in synaptophysin resulting from LPS treatment alone (*p = 0.019) and a subsequent treatment with clodronate was able to prevent this significant difference (*p* = 0.24) (Fig. 2k). Unlike with clodronate pre-treatment, however, there was no significant rescue when comparing LPS-treated cultures with and without clodronate (*p* = 0.11). Thus, treatment after the inflammatory insult is partially effective but less so than pre-emptive microglial depletion.

### IL1β is increased by LPS and is sufficient to induce synaptophysin depletion in OHSCs

**Fig. 3:**
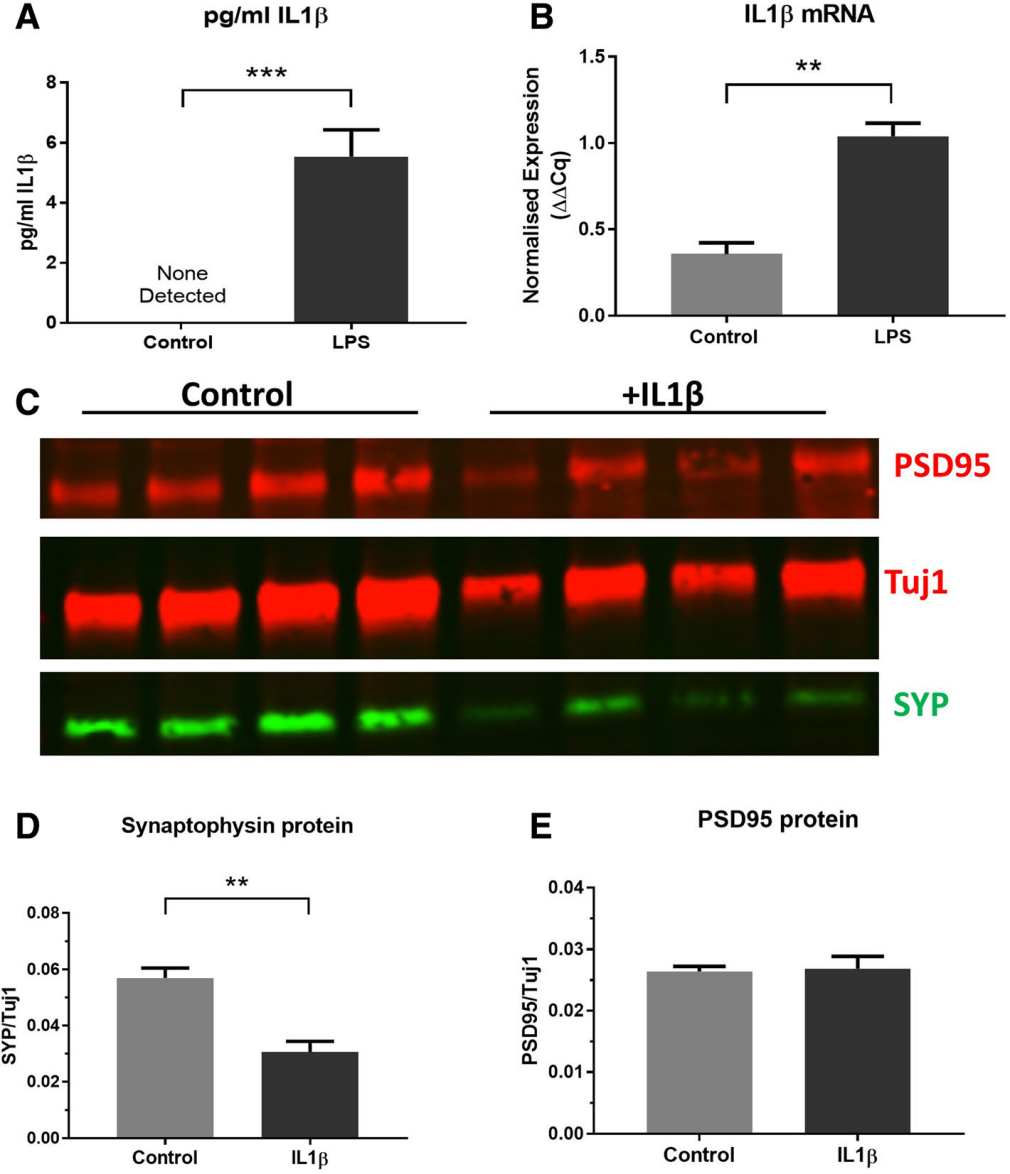
IL1β production is induced by LPS and addition of IL1β results in synaptophysin loss. LPS-treated OHSCs show increased IL1β protein levels in the culture medium after 24 h (***p = 0.0008) (*n* = 7) (**a**). IL1β mRNA is also elevated in 3 weeks in vitro wild-type cultures treated with LPS for the last week in vitro (**p = 0.0069) (*n* = 4) (**b**). Treatment with 20 ng/ml IL1β (**c**) results in reduced synaptophysin protein (**p = 0.0081) (**d**) with no significant change in PSD95 (*p* = 0.86) (**e**) (*n* = 4). All statistics were conducted using a paired *t* test to account for matched control and treated OHSCs from the same animal. Error bars = mean ± SEM

To determine whether a direct action of LPS on brain tissue causes loss of synaptophysin protein through release of inflammatory cytokines, the concentration of IL1β in the OHSC medium was determined by ELISA. Whilst in the untreated culture medium, the levels of IL1β were undetectable, in LPS-treated cultures, there was an average of 6 pg/ml IL1β detected, representing a highly significant increase (***p = 0.0008) (Fig. 3a). This observation occurs alongside a significant increase in IL1β mRNA in the slice tissue (**p = 0.0069) (Fig. 3b) indicating increased transcription of this inflammatory cytokine. To test whether such downstream production of IL1β is sufficient to deplete synaptophysin, murine IL1β protein was applied directly to OHSCs at 14 days in vitro. After 7 days of 20 ng/ml IL1β, OHSCs were harvested for western blot analysis (Fig. 3c). As with LPS treatment (see Fig. 1 and Fig. 2), there is a significant decrease in synaptophysin relative to Tuj1 (**p = 0.0081) (Fig. 3d) and no significant change in PSD95 (*p* = 0.86) (Fig. 3e). This demonstrates that IL1β is secreted from brain-resident cells after LPS treatment and is *sufficient* to induce synaptophysin loss in OHSCs.

### Microglia-derived IL1β plays a key role in synaptophysin depletion after LPS treatment

**Fig. 4:**
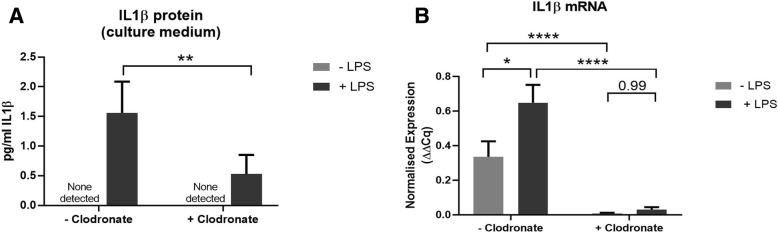
Depletion of microglia lowers IL1β mRNA and protein. Application of LPS to OHSCs results in a significant increase in IL1β protein in the culture medium (overall effect of LPS *p = 0.019) which is lowered in cultures pre-treated with clodronate (**p = 0.0025) (*n* = 5–6 per treatment group) (**a**). IL1β mRNA transcript is significantly upregulated in clodronate-naïve OHSCs after LPS treatment (*p = 0.02), but pre-treatment with clodronate lowers transcript levels to almost undetectable levels (****p < 0.0001) (*n* = 4–6 per treatment group) (**b**). Analysis was conducted using two-way ANOVA. Error bars = mean ± SEM

To determine whether the rise in IL1β detected after LPS treatment originated from microglia, IL1β protein (Fig. 4a) and mRNA (Fig. 4b) levels were analysed in cultures treated with LPS and/or clodronate (as for Fig. 2). Whilst IL1β was undetectable in the culture medium of LPS-naïve OHSCs, LPS treatment resulted in detectable levels of this cytokine (Fig. 4a) (*p = 0.019). Pre-treatment with clodronate resulted in a significant reduction in IL1β protein in LPS-treated cultures (**p = 0.0025). Similarly, IL1β mRNA transcript was significantly increased after LPS treatment in clodronate-naïve OHSCs (*p = 0.02), with clodronate pre-treatment significantly lowering transcript levels to almost undetectable levels (****p < 0.0001) (Fig. 4b). Taken together, the preservation of synaptophysin levels seen when microglia are pre-emptively depleted prior to LPS treatment (Fig. 2) shows a strong association with the levels of IL1β at both the protein and mRNA level.

**Fig. 5:**
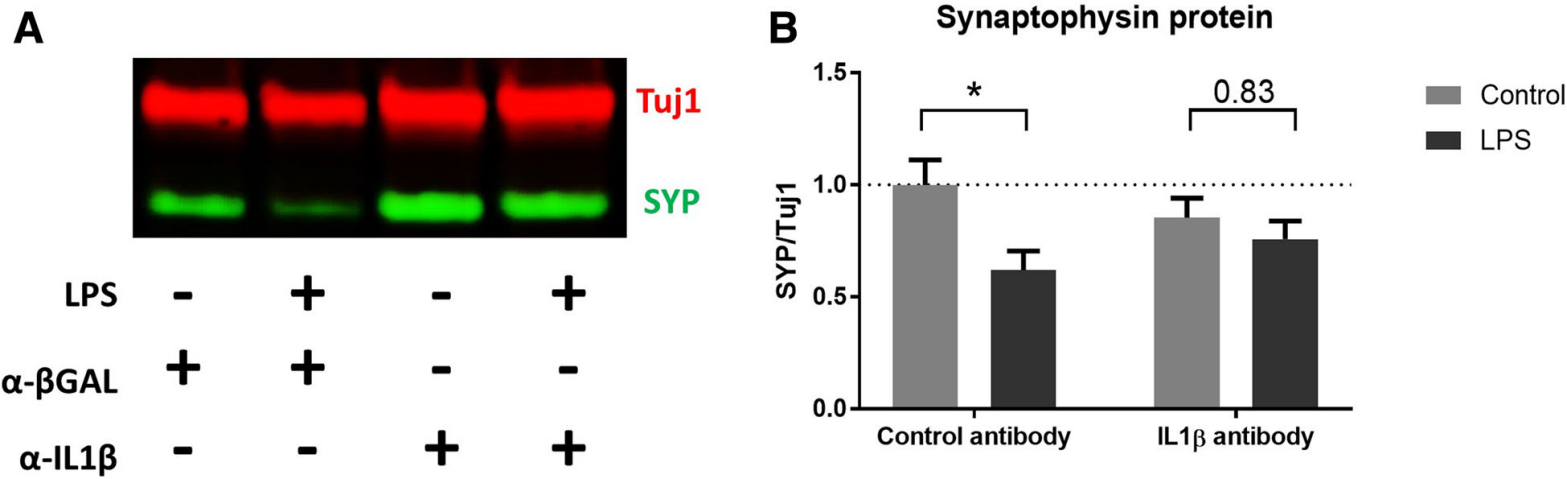
Application of anti-IL1β-neutralising antibody alters the OHSC response to LPS. Western blot of antibody and LPS-treated cultures (**a**) shows that whilst OHSCs pre-treated with anti-βGAL (control) antibody show a reduction in synaptophysin when treated with LPS (*p = 0.02) cultures pre-treated with anti-IL1β-neutralising antibody are resistant to LPS addition (*p* = 0.83) (**b**). There is no significant rescue of synaptophysin levels when comparing control antibody +LPS and anti-IL1β antibody + LPS (*p* = 0.63). All analysis was conducted using a two-way ANOVA (*n* = 16 per treatment group). Error bars = mean ± SEM

To determine whether IL1β is *necessary* for LPS to induce synaptophysin loss, 13 days in vitro OHSCs were treated with either a murine IL1β-neutralising mouse monoclonal antibody (α-IL1β) or a mouse IgG isotype control antibody specific to *E. coli* β-galactosidase (α-βGAL). Twenty-four hours later, cultures were treated with 200 ng/ml LPS. OHSCs were prepared such that the four different treatment conditions could be compared in tissue from the same animal. Slices were harvested at 21 days in vitro (7 days after LPS treatment) and analysed by western blot (Fig. 5a). Whilst OHSCs treated with the isotype control antibody showed the expected loss of synaptophysin protein in response to LPS treatment (*p = 0.02), cultures treated with IL1β-neutralising antibody were not sensitive to the addition of LPS (*p* = 0.83) (Fig. 5b). There was, however, no significant rescue when comparing LPS-exposed cultures treated with control versus IL1β-neutralising antibody (*p* = 0.63), although this is partly explained by a lowering of baseline synaptophysin levels by IL1β-neutralising antibody.

### Assessing synaptic protein recovery after LPS removal

**Fig. 6:**
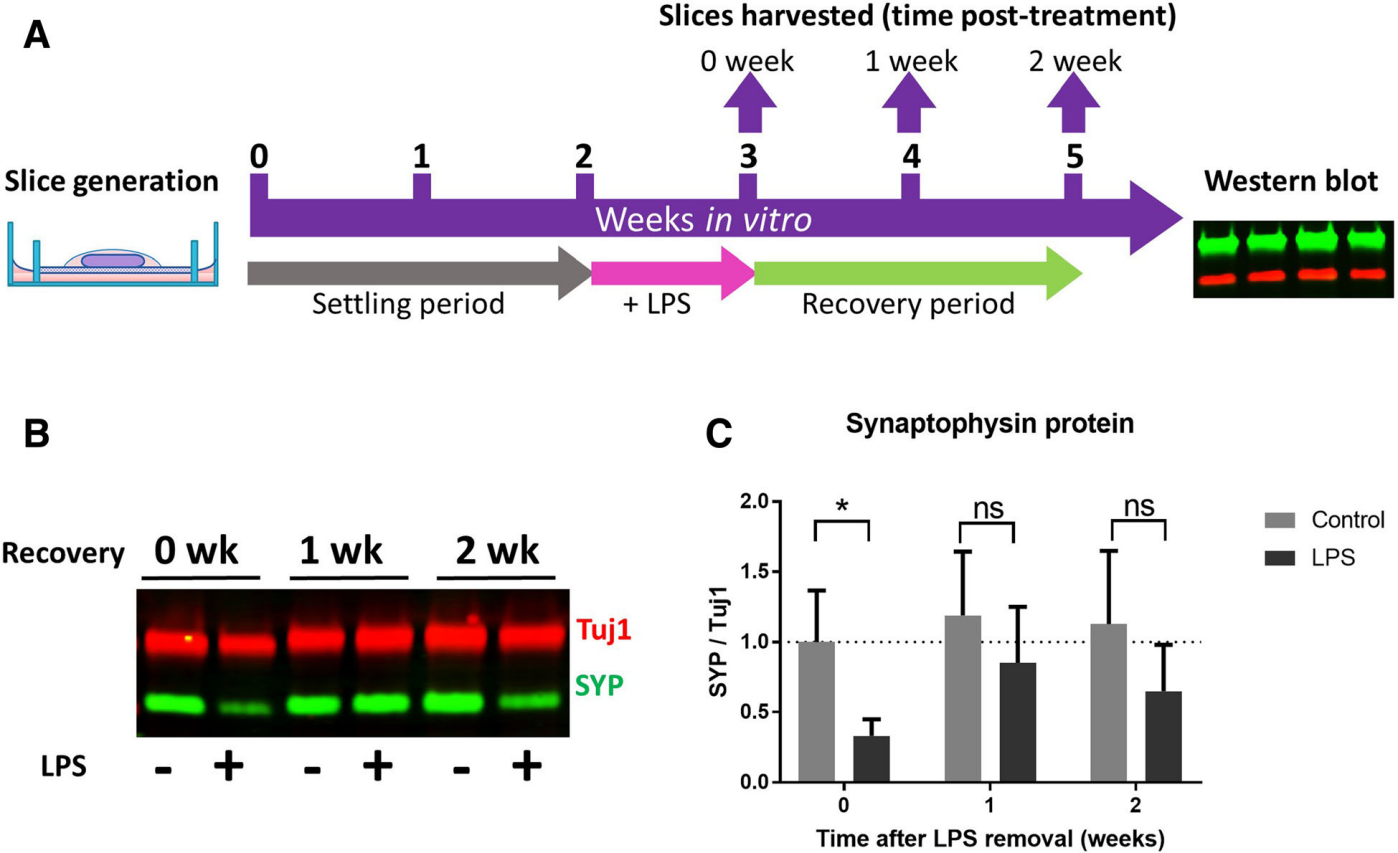
Synaptophysin protein levels undergo recovery after LPS removal. A diagrammatic representation of the LPS recovery experiment (**a**). OHSCs are aged for 2 weeks in vitro before undergoing 1 week of 200 ng/ml LPS. At 3 weeks in vitro, some slices are harvested to represent a 0 week after LPS removal timepoint. All other cultures undergo a 100% medium exchange to LPS-free medium. Slices are then harvested at either 1 week or 2 weeks after LPS removal and synaptophysin protein levels assessed by western blot (**b**). Slices harvested with no recovery after LPS removal showed a reduction in synaptophysin levels when compared to untreated samples (*p = 0.014). At 1 week (*p* = 0.30) or 2 weeks (*p* = 0.13) after LPS removal, there is no significance between LPS-exposed and untreated OHSCs (**c**). Analysis was conducted using a two-way ANOVA (*n* = 5–6 per timepoint and treatment group). Error bars = mean ± SEM

Next, we sought to determine whether complete removal of the inflammatory stimulus (LPS) permits recovery of synaptic protein to untreated levels. This models responses that may be seen clinically when prophylactic treatment is not an option. Complete and rapid removal of inflammatory stimuli from the brain is particularly difficult to ensure in vivo, but manipulation of the extracellular environment in this way is straightforward in OHSCs*.* OHSCs were prepared such that two culture dishes were created per mouse (each with three slices). After 2 weeks in vitro, all of the cultures underwent a 100% medium exchange, with one of the two dishes receiving 200 ng/ml LPS. After a further week of treatment, one group of slices (those representing a 0-week post-treatment timepoint) was harvested for western blot whilst all other cultures underwent a further 100% medium exchange, receiving untreated medium. Slices were then left to “recover” for a further 1 or 2 weeks in vitro (Fig. 6a). Synaptophysin protein levels in OHSC lysates were analysed by western blot (Fig. 6b). As expected, OHSCs harvested immediately after LPS treatment showed a reduction in synaptophysin protein when compared to untreated slices from the same animal (*p = 0.014) (Fig. 6c). However, synaptophysin levels were no longer significantly different in treated vs untreated OHSCs at 1 week (*p* = 0.30) or 2 weeks (*p* = 0.13) after LPS washout. This indicates that loss of synaptophysin in response to LPS is substantially reversible after the inflammatory insult is removed, although the prospect of a more complete recovery over a longer timescale is difficult to study in OHSCs due to gradual divergence from tissue in vivo.

### LPS does not cause synaptophysin loss through alterations to the amyloid pathway

**Fig. 7:**
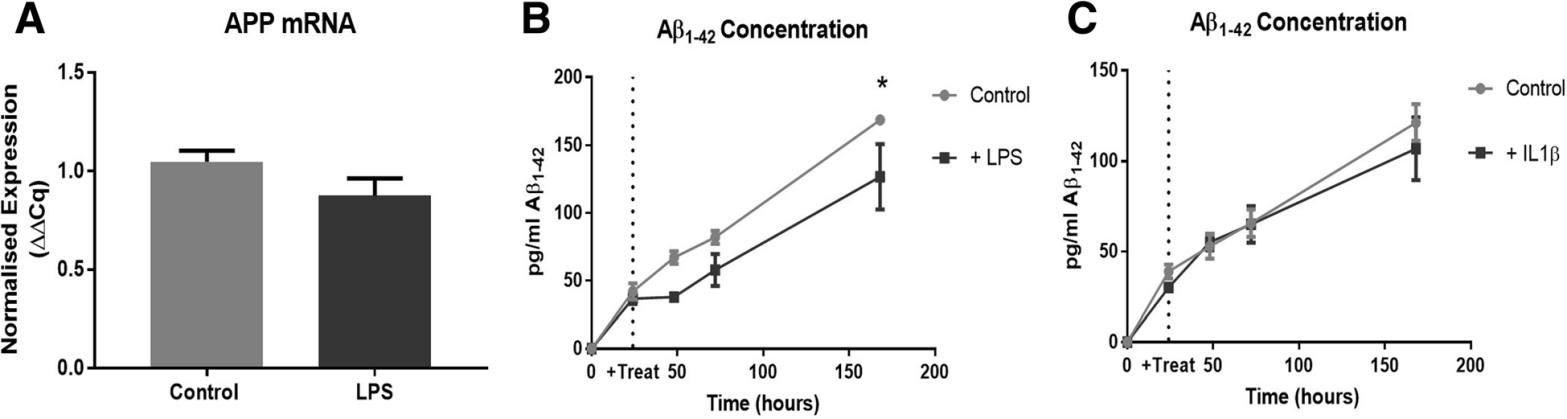
LPS does not interact with the amyloid pathway in OHSCs. LPS treatment does not alter APP mRNA expression levels (*p* = 0.20) (*n* = 7) (**a**). ELISA analysis of OHSC medium reveals that LPS treatment reduces the production of Aβ_1–42_ (two-way ANOVA: effect of treatment *p* = 0.053 (*n* = 4)) (**b**) whilst IL1β application does not influence Aβ_1–42_ accumulation (two-way ANOVA effect of treatment *p* = 0.56 (*n* = 4)) (**c**). Error bars = mean ± SEM

Finally, as inflammation is often linked to increased cognitive decline in people living with dementia [8], and levels of soluble Aβ have been strongly correlated with synapse loss [54], we tested the hypothesis that the effects of LPS on wild-type OHSCs could interact with Alzheimer’s disease pathogenic mechanisms. We saw no change in APP mRNA in wild-type OHSCs treated with LPS (*p* = 0.20) (Fig. 7a) and neither treatment with LPS (*p* = 0.053) (Fig. 7b) nor IL1β (*p* = 0.56) (Fig. 7c) increased the production of murine Aβ_1–42_ (as measured by protein concentration in the culture medium). Indeed, at later timepoints, LPS treatment significantly *reduced* the detectable level of Aβ in the culture medium. This demonstrates that, in the OHSC system, loss of synaptophysin is not induced by alterations in Aβ production.

## Discussion

Understanding neuroinflammatory mechanisms of synapse loss is crucial for the development of effective therapeutics for a wide range of neurodegenerative disorders, but there are significant challenges to addressing such mechanisms within the brain. Using isolated brain tissue maintained for many weeks in culture, we report a mechanism dependent on microglia and involving IL1β activity. Loss of synaptophysin occurs in the absence of significant cell death, alterations in APP or Aβ production and prior to any change in PSD95 protein levels. These data confirm, for the first time, that inflammatory signalling taking place entirely within brain tissue can lead to presynaptic disruption, and we present an important new experimental model for further exploration of mechanisms and therapy.

Our finding that LPS administration results in the loss of synaptophysin confirms and extends findings previously reported *in vivo* [16, 17]. Whilst we do not see alterations to PSD95 protein under our experimental conditions, a prior study found application of LPS (at five times the concentration used here) induced dendritic spine loss in OHSCs [55]. Our finding that there is a reduction in synaptophysin and PSD95 mRNA could indicate that whilst both compartments are sensitive to disruption by LPS, presynaptic protein disruption occurs earlier and under lower levels of inflammatory insult, potentially due to differences in protein turnover rates. Subsequent loss of dendritic spines could then be a consequence of deafferentation. These authors do not report having examined the presynaptic compartment in their work, so it is feasible that both a pre- and post-synaptic deficit is present in their model. Likewise, the presence of PSD95 in a western blot does not prove that this protein exists in structurally normal spines so we cannot rule out changes to the organisation of the post-synaptic compartment in our work.

Taking advantage of the versatility of this model, we were able to show that depleting microglia in the absence of any peripheral cell types, prior to LPS insult, prevents the loss of synaptophysin. This adds considerable weight to the hypothesis that microglia, and not infiltrating immune cells from the periphery, are the mediators of LPS-induced synaptic damage. Specific depletion of microglia in this system was possible due to the accessibility of OHSCs to pharmacological manipulation and simplified by the isolation from peripheral circulating immune cells, whose depletion may induce confounding effects that could mask the impact on the brain-resident microglia [30]. We also explored whether microglial depletion 24 h after LPS administration could alter the synaptic response, more closely mimicking potential treatments, where there may be a delay in identifying the onset of inflammation. This also prevented a significant drop in synaptophysin levels when compared to LPS-naïve OHSCs, but without significant improvement relative to cultures treated with LPS alone. This indicates that rapid intervention may be required to completely prevent synapse loss in response to microglial activation, but some efficacy may be retained at later timepoints.

Our work here supports a pivotal role for IL1β in inducing hippocampal synaptophysin loss in response to neuroinflammatory insult. We show, for the first time, that direct application of IL1β to isolated brain tissue results in loss of synaptic proteins, mimicking the effect of LPS addition. Work in acute hippocampal slice cultures has previously shown that IL1β disrupts synaptic transmission, abrogates LTD and inhibits LTP [56–59], but we now extend this to longer term effects on the protein level. This is important because studies in acute hippocampal slices take place in highly inflammatory conditions activated by the slicing injury, which we avoid by allowing a 2-week settling period prior to experimentation. Thus, even when basal inflammation is low, IL1β disrupts synapses.

We demonstrate that IL1β at both the protein and mRNA levels are increased in OHSCs in response to LPS and this effect is prevented when microglia are depleted using clodronate. IL1β-neutralising antibody applied prior to, and throughout, LPS treatment partially rescues the levels of synaptophysin. Unlike microglial depletion, the neutralisation of IL1β (and/or other cytokines) is a feasible strategy to protect synapses after an inflammatory insult in a clinical setting. As the production of IL1β increases after LPS addition, and can readily be detected by ELISA, it may be possible to test patient blood or CSF samples after a potential inflammatory insult to assess whether synaptic damage is likely to occur and treat accordingly. As we did not see a complete rescue of synaptophysin levels when comparing LPS-treated cultures with control versus IL1β-neutralising antibody, further investigation into additional mediators of synaptophysin loss may be required. A combinatorial treatment targeting multiple inflammatory cytokines might prove a more effective strategy and could be readily trialled in this system.

Whilst cultures treated with IL1β-neutralising antibody were resistant to the effects of LPS on synaptophysin levels, there appeared to be a small effect on basal synaptic protein levels, which could be responsible for partial rescue phenotype seen. Interestingly, a role of IL1β in regulating *physiological* synaptic processes has been previously proposed, with studies demonstrating hippocampal enrichment of IL-1 receptors, enhancement of IL1β production after LTP and cognitive deficits induced by IL-1 receptor knockout or IL-1 receptor antagonist administration to otherwise healthy mice [21, 60–62]. It could be that application of IL1β-neutralising antibody may itself impact baseline synaptic processes and should be considered when designing therapeutics to target IL1β in neuroinflammation.

Our finding that complete washout of LPS (made possible by the accessibility of the OHSC system to environmental manipulation) permits recovery of synaptophysin levels to approaching that of untreated cultures indicates that at least partial synaptic recovery is possible if the inflammatory insult is removed. Whilst studies in human patients commonly report long-term cognitive deficits or worsening of neurodegenerative disease processes after acute inflammatory insults [4], it is often seen that these patients retain high levels of circulating inflammatory cytokines [1]. Devising treatments, such as those targeting IL1β that could “reset” the extracellular environment to a non-inflammatory state, could be of benefit when seeking to prevent cognitive decline.

Finally, we observe that addition of LPS does not alter mRNA levels of APP or the production of Aβ in OHSCs. In fact, Aβ secretion, which is readily testable in this system, is significantly inhibited over time, consistent with previous reports that microglia activation results in increased clearance of diffuse Aβ [26–29]. Whilst some studies report increases in Aβ in response to neuroinflammatory stimuli [20], the use of different experimental models and variation in methods of inflammatory treatment prevent direct comparison. It seems likely that inflammatory and neurodegenerative disease processes interact in complex ways and there may be multiple independent or converging mechanisms that result in synaptic disruption. Loss of synapses in the brain is widely reported to correlate with clinical outcome in a range of neurodegenerative diseases including Alzheimer’s disease [63–65], amyotrophic lateral sclerosis [66] and frontotemporal dementia [67]. Understanding mechanisms by which synapses are lost, and developing therapies to protect these vital structures, is therefore a key priority when seeking to develop disease-altering therapeutics.

## Conclusions

In summary, we have shown that the addition of LPS directly to isolated brain tissue results in loss of the presynaptic protein synaptophysin in the absence of significant cell death, changes to PSD95 protein levels or Aβ production. The depletion of synaptic proteins can be prevented by pre-treatment with the microglia-specific toxin clodronate *prior to*, and to a lesser extent *after*, LPS exposure, and recovery begins spontaneously after rapid and complete LPS removal. There is a microglial-dependent upregulation of IL1β mRNA and protein after LPS treatment, and this is sufficient to induce significant loss of synaptophysin. Application of IL1β-neutralising antibody protects against the effects of LPS but may impact basal synaptic processes. By exploring mechanisms of neuroinflammatory synapse loss in a model system that mitigates unavoidable limitations of primary culture and in vivo work, our study strengthens the evidence for a key role of microglia-derived IL1β in inducing synaptic dysfunction after inflammatory insult.

## Additional file

Additional file 1: **Figure S1.** Treatment with LPS and clodronate does not result in significant cell death. (a–d) OHSCs were live-stained with propidium iodide 7 days after LPS treatment (at 21 days in vitro). There was no significant cell death in control (a), LPS (b), clodronate treated (c) or LPS + clodronate (d) treated OHSCs. As a positive control, OHSCs treated with 100% ethanol for 5 min showed extensive propidium iodide labelling indicating mass cell death (e). (JPG 244 kb)

## Abbreviations

APP: Amyloid precursor protein
Aβ: Amyloid beta peptide
ELISA: Enzyme-linked immunosorbent assay
IL1β: Interleukin 1β
LPS: Lipopolysaccharide
LTD: Long-term depression
LTP: Long-term potentiation
OHSC: Organotypic hippocampal slice culture
PBS: Phosphate-buffered saline
PBS-T: Phosphate-buffered saline with 0.1% Tween-20
PSD95: Post-synaptic density 95
SYP: Synaptophysin

## Author’s information

Claire Durrant was previously known as Claire Harwell.

## References

[1] Yende S (2008). Inflammatory markers at hospital discharge predict subsequent mortality after pneumonia and sepsis. Am J Respir Crit Care Med.

[2] Ide M (2016). Periodontitis and cognitive decline in Alzheimer’s disease. PLoS One.

[3] Annane D, Sharshar T (2015). Cognitive decline after sepsis. Lancet Respir Med.

[4] Iwashyna TJ, Ely EW, Smith DM, Langa KM (2010). Long-term cognitive impairment and functional disability among survivors of severe sepsis. JAMA.

[5] Benros ME (2015). The association between infections and general cognitive ability in young men – a nationwide study. PLoS One.

[6] Engelhart MJ (2004). Inflammatory proteins in plasma and the risk of dementia: the Rotterdam study. Arch Neurol.

[7] Tsai C-H, et al. Fracture as an Independent Risk Factor of Dementia: A Nationwide Population-Based Cohort Study. Medicine (Baltimore). 2014;93:e188. 10.1097/MD.0000000000000188 PMC4616393 25474435

[8] Holmes C (2009). Systemic inflammation and disease progression in Alzheimer disease. Neurology.

[9] Zotova E, Nicoll JA, Kalaria R, Holmes C, Boche D (2010). Inflammation in Alzheimer’s disease: relevance to pathogenesis and therapy. Alzheimers Res Ther.

[10] Akiyama H (2000). Inflammation and Alzheimer’s disease. Neurobiol Aging.

[11] Buljevac D (2002). Prospective study on the relationship between infections and multiple sclerosis exacerbations. Brain.

[12] Perry VH, Cunningham C, Holmes C (2007). Systemic infections and inflammation affect chronic neurodegeneration. Nat Rev Immunol.

[13] Umemura A (2014). Delirium and high fever are associated with subacute motor deterioration in Parkinson disease: a nested case-control study. PLoS One.

[14] Andersson PB, Perry VH, Gordon S (1992). The acute inflammatory response to lipopolysaccharide in CNS parenchyma differs from that in other body tissues. Neuroscience.

[15] Mina F (2014). Il1-β involvement in cognitive impairment after sepsis. Mol Neurobiol.

[16] Moraes CA (2015). Activated microglia-induced deficits in excitatory synapses through IL-1β: implications for cognitive impairment in sepsis. Mol Neurobiol.

[17] Deng X-H (2012). Lipopolysaccharide induces paired immunoglobulin-like receptor B (PirB) expression, synaptic alteration, and learning–memory deficit in rats. Neuroscience.

[18] Han Q (2017). Microglia-derived IL-1β contributes to axon development disorders and synaptic deficit through p38-MAPK signal pathway in septic neonatal rats. J Neuroinflammation.

[19] Hasegawa-Ishii S, Shimada A, Imamura F (2017). Lipopolysaccharide-initiated persistent rhinitis causes gliosis and synaptic loss in the olfactory bulb. Sci Rep.

[20] Lee JW (2008). Neuro-inflammation induced by lipopolysaccharide causes cognitive impairment through enhancement of beta-amyloid generation. J Neuroinflammation.

[21] Prieto GA (2015). Synapse-specific IL-1 receptor subunit reconfiguration augments vulnerability to IL-1β in the aged hippocampus. Proc Natl Acad Sci.

[22] Shaw KN, Commins S, O’Mara SM (2001). Lipopolysaccharide causes deficits in spatial learning in the watermaze but not in BDNF expression in the rat dentate gyrus. Behav Brain Res.

[23] Terrando N (2010). The impact of IL-1 modulation on the development of lipopolysaccharide-induced cognitive dysfunction. Crit Care.

[24] Sheng JG (2003). Lipopolysaccharide-induced-neuroinflammation increases intracellular accumulation of amyloid precursor protein and amyloid β peptide in APPswe transgenic mice. Neurobiol Dis.

[25] Ma L (2016). TSPO ligand PK11195 alleviates neuroinflammation and beta-amyloid generation induced by systemic LPS administration. Brain Res Bull.

[26] Go M, Kou J, Lim J-E, Yang J, Fukuchi K (2016). Microglial response to LPS increases in wild-type mice during aging but diminishes in an Alzheimer’s mouse model: implication of TLR4 signaling in disease progression. Biochem Biophys Res Commun.

[27] Herber DL (2004). Time-dependent reduction in Aβ levels after intracranial LPS administration in APP transgenic mice. Exp Neurol.

[28] Herber DL (2007). Microglial activation is required for Abeta clearance after intracranial injection of lipopolysaccharide in APP transgenic mice. J. Neuroimmune Pharmacol.

[29] DiCarlo G, Wilcock D, Henderson D, Gordon M, Morgan D (2001). Intrahippocampal LPS injections reduce Abeta load in APP+PS1 transgenic mice. Neurobiol Aging.

[30] Han J, Harris RA, Zhang X-M (2017). An updated assessment of microglia depletion: current concepts and future directions. Mol Brain.

[31] Bobula B, Sowa J, Hess G (2015). Anti-interleukin-1β antibody prevents the occurrence of repeated restraint stress-induced alterations in synaptic transmission and long-term potentiation in the rat frontal cortex. Pharmacol Rep.

[32] Poduslo JF, Curran GL, Berg CT (1994). Macromolecular permeability across the blood-nerve and blood-brain barriers. Proc Natl Acad Sci.

[33] Li Y, Liu L, Barger SW, Griffin WST (2003). Interleukin-1 mediates pathological effects of microglia on tau phosphorylation and on synaptophysin synthesis in cortical neurons through a p38-MAPK pathway. J Neurosci.

[34] Mishra A, Kim HJ, Shin AH, Thayer SA (2012). Synapse loss induced by interleukin-1β requires pre- and post-synaptic mechanisms. J Neuroimmune Pharmacol.

[35] Yang S (2005). Interleukin-1β enhances NMDA receptor-mediated current but inhibits excitatory synaptic transmission. Brain Res.

[36] Araque A, Parpura V, Sanzgiri RP, Haydon PG (1999). Tripartite synapses: glia, the unacknowledged partner. Trends Neurosci.

[37] Perea G, Navarrete M, Araque A (2009). Tripartite synapses: astrocytes process and control synaptic information. Trends Neurosci.

[38] Zagrebelsky M, Schweigreiter R, Bandtlow CE, Schwab ME, Korte M (2010). Nogo-A stabilizes the architecture of hippocampal neurons. J Neurosci.

[39] Druckmann S (2014). Structured synaptic connectivity between hippocampal regions. Neuron.

[40] Allen NJ, Eroglu C (2017). Cell biology of astrocyte-synapse interactions. Neuron.

[41] Chung W-S, Allen NJ, Eroglu C (2015). Astrocytes control synapse formation, function, and elimination. Cold Spring Harb Perspect Biol.

[42] Vitureira N, Letellier M, Goda Y (2012). Homeostatic synaptic plasticity: from single synapses to neural circuits. Curr Opin Neurobiol.

[43] Miyamoto A (2016). Microglia contact induces synapse formation in developing somatosensory cortex. Nat Commun.

[44] Kato G, et al. Microglial Contact Prevents Excess Depolarization and Rescues Neurons from Excitotoxicity. eneuro. 2016;3:ENEURO.0004–16.2016. 10.1523/ENEURO.0004-16.2016 PMC4916329 27390772

[45] Wu Y, Dissing-Olesen L, MacVicar BA, Stevens B (2015). Microglia: dynamic mediators of synapse development and plasticity. Trends Immunol.

[46] Kettenmann H, Kirchhoff F, Verkhratsky A (2013). Microglia: new roles for the synaptic stripper. Neuron.

[47] Holm TH, Draeby D, Owens T (2012). Microglia are required for astroglial toll-like receptor 4 response and for optimal TLR2 and TLR3 response. Glia.

[48] De Simoni A, MY Yu L (2006). Preparation of organotypic hippocampal slice cultures: interface method. Nat Protoc.

[49] Harwell CS, Coleman MP (2016). Synaptophysin depletion and intraneuronal Aβ in organotypic hippocampal slice cultures from huAPP transgenic mice. Mol Neurodegener.

[50] Croft CL, Noble W (2018). Preparation of organotypic brain slice cultures for the study of Alzheimer’s disease. F1000Res.

[51] Humpel C (2015). Organotypic brain slice cultures: a review. Neuroscience.

[52] Kohl A, Dehghani F, Korf H-W, Hailer NP (2003). The bisphosphonate clodronate depletes microglial cells in excitotoxically injured organotypic hippocampal slice cultures. Exp Neurol.

[53] Kumamaru H (2012). Liposomal clodronate selectively eliminates microglia from primary astrocyte cultures. J Neuroinflammation.

[54] Lue L-F (1999). Soluble amyloid β peptide concentration as a predictor of synaptic change in Alzheimer’s disease. Am J Pathol.

[55] Chang PK-Y, Khatchadourian A, McKinney RA, Maysinger D (2015). Docosahexaenoic acid (DHA): a modulator of microglia activity and dendritic spine morphology. J Neuroinflammation.

[56] Yu B, Shinnick-Gallagher P (1994). Interleukin-1 beta inhibits synaptic transmission and induces membrane hyperpolarization in amygdala neurons. J Pharmacol Exp Ther.

[57] Ikegaya Y, Delcroix I, Iwakura Y, Matsuki N, Nishiyama N (2003). Interleukin-1beta abrogates long-term depression of hippocampal CA1 synaptic transmission. Synap N Y N.

[58] Cunningham AJ, Murray CA, O’Neill LAJ, Lynch MA, O’Connor JJ (1996). Interleukin-1β (IL-1β) and tumour necrosis factor (TNF) inhibit long-term potentiation in the rat dentate gyrus in vitro. Neurosci Lett.

[59] Bellinger FP, Madamba S, Siggins GR (1993). Interleukin 1β inhibits synaptic strength and long-term potentiation in the rat CA1 hippocampus. Brain Res.

[60] Huang Z-B, Sheng G-Q (2010). Interleukin-1β with learning and memory. Neurosci Bull.

[61] Schneider H (1998). A neuromodulatory role of interleukin-1β in the hippocampus. Proc Natl Acad Sci.

[62] Levin SG, Godukhin OV (2017). Modulating effect of cytokines on mechanisms of synaptic plasticity in the brain. Biochem Mosc.

[63] Terry RD (1991). Physical basis of cognitive alterations in Alzheimer’s disease: synapse loss is the major correlate of cognitive impairment. Ann Neurol.

[64] Scheff SW, Price DA, Schmitt FA, DeKosky ST, Mufson EJ (2007). Synaptic alterations in CA1 in mild Alzheimer disease and mild cognitive impairment. Neurology.

[65] DeKosky ST, Scheff SW (1990). Synapse loss in frontal cortex biopsies in Alzheimer’s disease: correlation with cognitive severity. Ann Neurol.

[66] Henstridge CM (2018). Synapse loss in the prefrontal cortex is associated with cognitive decline in amyotrophic lateral sclerosis. Acta Neuropathol (Berl).

[67] Lipton AM (2001). Contribution of asymmetric synapse loss to lateralizing clinical deficits in frontotemporal dementias. Arch Neurol.

