# Supplementary figures and images for: Lipopolysaccharide-induced neuroinflammation induces presynaptic disruption through a direct action on brain tissue involving microglia-derived interleukin 1 beta
Source: J Neuroinflammation. 2019 May 18;16:106. doi: 10.1186/s12974-019-1490-8 (PMC6525970; doi:10.1186/s12974-019-1490-8)

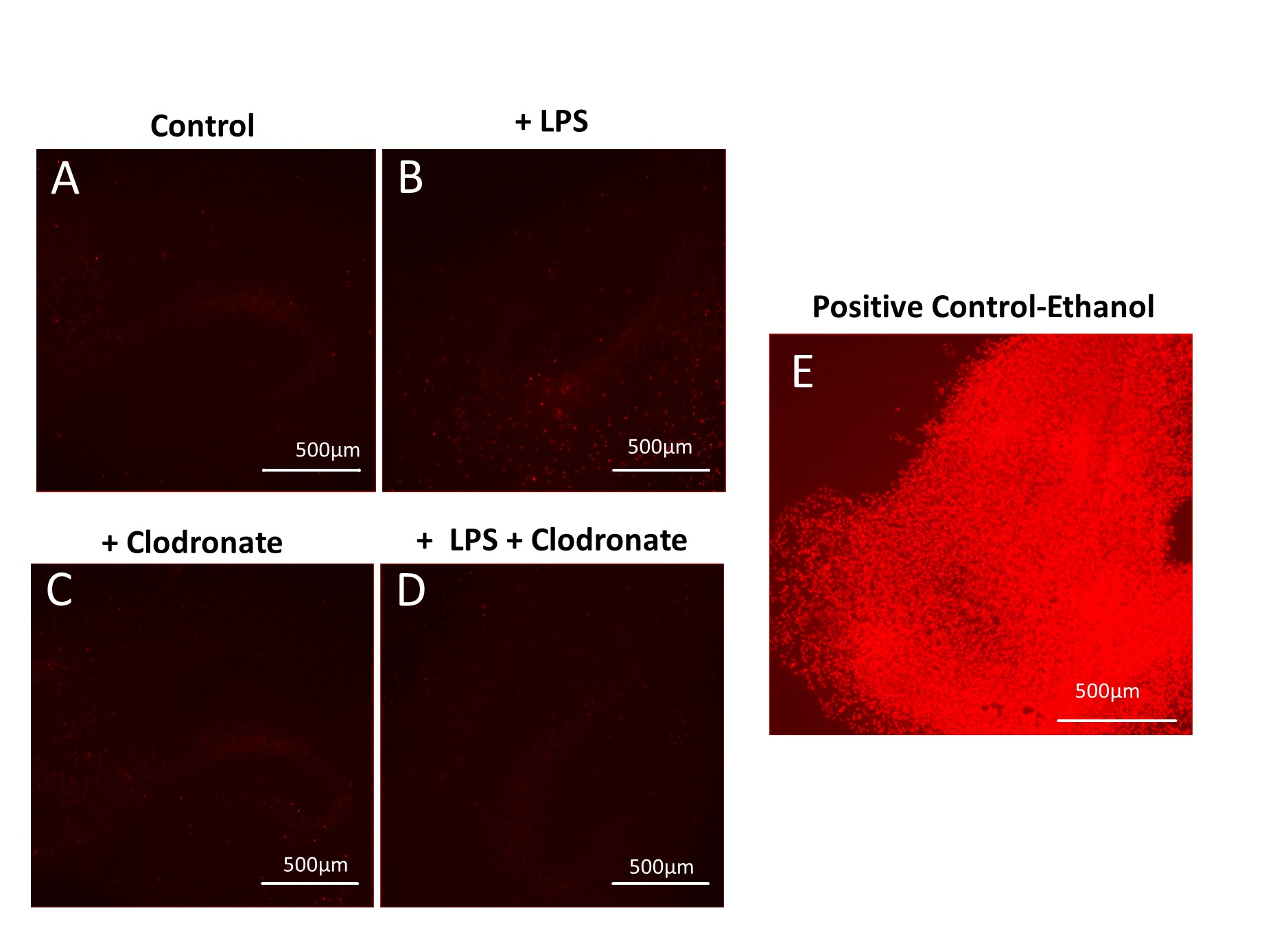

Supplement: Supplementary file 1 — Figure S1. Treatment with LPS and clodronate does not result in significant cell death. (a–d) OHSCs were live-stained with propidium iodide 7 days after LPS treatment (at 21 days in vitro). There was no significant cell death in control (a), LPS (b), clodronate treated (c) or LPS + clodronate (d) treated OHSCs. As a positive control, OHSCs treated with 100% ethanol for 5 min showed extensive propidium iodide labelling indicating mass cell death (e). (JPG 244 kb) [file 12974_2019_1490_MOESM1_ESM.jpg]
